# Validation of a urine color scale for assessment of urine osmolality in healthy children

**DOI:** 10.1007/s00394-015-0905-2

**Published:** 2015-04-24

**Authors:** Stavros A. Kavouras, Evan C. Johnson, Dimitris Bougatsas, Giannis Arnaoutis, Demosthenes B. Panagiotakos, Erica Perrier, Alexis Klein

**Affiliations:** Department of Health, Human Performance and Recreation, University of Arkansas, Fayetteville, AR 72701 USA; Department of Dietetics and Nutrition, Harokopio University, Athens, Greece; Danone Research, Palaiseau, France

**Keywords:** Hydration status, Children, Hydration assessment, Dehydration, Hypohydration markers

## Abstract

**Aim:**

Urine color (UC) is a practical tool for hydration assessment. The technique has been validated in adults, but has not been tested in children.

**Purpose:**

The purpose of the study was to test the validity of the urine color scale in young, healthy boys and girls, as a marker of urine concentration, investigate its diagnostic ability of detecting hypohydration and examine the ability of children to self-assess UC.

**Methods:**

A total of 210 children participated (age: 8–14 years, body mass: 43.4 ± 12.6 kg, height: 1.49 ± 0.13 m, body fat: 25.2 ± 7.8 %). Data collection included: two single urine samples (first morning and before lunch) and 24-h sampling. Hydration status was assessed via urine osmolality (UOsmo) and UC via the eight-point color scale.

**Results:**

Mean UC was 3 ± 1 and UOsmo 686 ± 223 mmol kg^−1^. UC displayed a positive relationship as a predictor of UOsmo (*R*^2^: 0.45, *P* < 0.001). Based on the receiver operating curve, UC has good overall classification ability for the three samples (area under the curve 85–92 %), with good sensitivity (92–98 %) and specificity (55–68 %) for detecting hypohydration. The overall accuracy of the self-assessment of UC in the morning or the noon samples ranged from 67 to 78 %. Further threshold analysis indicated that the optimal self-assessed UC threshold for hypohydration was ≥4.

**Conclusions:**

The classical eight-point urine color scale is a valid method to assess hydration in children of age 8–14 years, either by researchers or self-assessment.

## Introduction

Adequate fluid intake and hydration status is important to sports performance, cognition and overall well-being in adults [1–4], and children [5–7]. Within large-scale investigations, urinary markers, specifically osmolality (UOsmo), have been identified as an easy-to-use, laboratory-based, hydration assessment technique [8]. When a more expedient hydration status assessment is beneficial, urine color (UC) has been shown to be a practical tool for assessment of urine concentration and changes body water in both field and laboratory settings [9, 10]. The ease-of-use of UC makes it an attractive method for athletic trainers, clinicians and lay individuals seeking to evaluate their day-to-day hydration and attain optimal fluid consumption behaviors prior to athletic endeavors and over the course of normal daily living. However, the sensitivity of UC as a clinical measure of hydration status has been brought up as a potential limiting factor to its use [8]. Additionally, UC use in children is limited because previous validation investigations established its utility through measurements within the adult population. Yet the implications of hydration status assessment within youth are equally as important, and thus validation across a wider age range is necessary.

Urine concentration, specifically a UOsmo ≥800 mmol kg^−1^, has been recommended by many previous investigations to denote hypohydration in populations of free-living adults and children outside of exercise-induced dehydration [6, 11–13]. In this context, hypohydration refers to alterations in the hydration process due to suboptimal fluid intake necessary for ideal excretion of solutes [14], combined with reduced cell hydration status and elevated levels of fluid conservatory hormones [15, 16]. Alternate interpretations of the term hypohydration, especially in the athletic arena, refer to either a loss of total body water or existence in a state of a lower than normal volume of total body water (i.e., hypertonic hypovolemia) [17, 18]. However, for investigations centered on measurement of, or physiological impacts due to differences in hydration process, the first definition is appropriate. As such, it is important to denote that references to hypohydration within the following investigation do not imply any change in or suboptimal volume of total body water.

UC is a viable marker of hydration status in adults, as evidenced by its strong positive relationship with UOsmo, as shown in the initial color scale investigations. However, UC is primarily due to the concentration of urochrome, which is a byproduct of hemoglobin breakdown [19], whereas UOsmo is primarily dependent on the osmotically active Na^+^, Cl^−^, K^+^ and urea molecules found in the urine [20]. Age affects relative red blood cell mass (mL RBC kg^−1^) [21, 22] and relative water recommendations (mL H_2_O kg^−1^) [23, 24], which results in different ratios of red blood cell mass to fluid intake between adults and children. Therefore, it is feasible that the relationship between UC and UOsmo observed in adults may differ in children because of altered urochrome output independent from urinary osmolyte excretion. Due to these physiological differences between the age groups, it is important that the UC to UOsmo relationship be validated within this specific population.

The need for valid hydration assessment within children is apparent because both US and European children have been observed to fall short of daily water recommendations [25–27]. Fortunately, it has been demonstrated that fluid interventions within children are effective at improving hydration status [10]. However, the collection and measurement of individual or 24-h urine samples by trained researchers is a labor- and time-intensive process. Additionally, requesting children to provide urine samples in a public setting can be a delicate topic. Therefore, it would be beneficial if children themselves were able to self-assess their hydration status. It has been shown that children are already perceptive of their hydration practices, but it does not carry over to changes in their hydration behaviors or hydration status [5]. The establishment of an acute measurement tool would give an anchor to allow children to be more aware of their hydration status and to improve hydration practices. Additionally, a valid hydration tool would simplify the implementation of future water interventions aimed at improving sub-standard hydration status that has been identified in some youth samples [13, 15, 27].

Lastly, in order for a hydration assessment to be effective, it is essential that the analyzed sample be indicative of the time period in question. In research situations, evaluation of a 24-h urine sample may be preferable to individual time-point urine samples because it accounts for fluctuations in urine concentration due to normal eating, drinking and exercise patterns [28]. However, collection of 24-h samples by adults or children in real-life conditions is not practical. Evidence exists that demonstrates “first-morning” urine samples are more concentrated than other points throughout the day which may conceal true assessment of hydration [29]. Perrier et al. [30] recently showed that early afternoon urine concentration is most similar to that of total 24-h urine collections, suggesting that this period may be appropriate as a surrogate sample to evaluate 24-h hydration status. For the reasons mentioned above, this relationship should also be confirmed in children.

Outside of hydration assessment prior to exercise and epidemiological studies of water intake, the data related to hydration assessment within children are limited. However, proper hydration within this population is relevant to overall health. Thus, we aimed to provide a comprehensive evaluation of a field-expedient UC hydration scale [10] with the current investigation. The aims were as follows: (a) to examine the validity of the urine color scale in young and healthy boys and girls as a marker of urine concentration against urine osmolality; (b) to investigate the diagnostic ability of urine color for hypohydration as defined by urine osmolality and identify the threshold value; and (c) to study ability of young healthy boys and girls to self-diagnose hypohydration based on the agreement of participant UC assessments and those of trained investigators. Examination of these aims will extend the use of the urine color scale and hydration assessment within youth.

## Experimental methods

### Subjects

A total of 210 healthy children (8–14 years old) selected from a convenient sample within the city of Athens, Greece, completed the study protocol between October 2012 and April 2013. The environmental temperature during data collection averaged 14.6 °C (5–24.5 °C).

Institutional review boards at Harokopio University and University of Arkansas approved the study. In accordance with the Declaration of Helsinki, all study procedures were explained to the children and participant’s legal guardian. Signed consent forms were obtained from each participant’s legal guardian, and the children provided verbal assent for participation.

A medical history questionnaire was used to exclude participants with conditions that could affect fluid balance. Full exclusionary criteria included the following: (a) presence of cardiovascular, hematological, hepatic, gastrointestinal, renal, pulmonary, endocrine or psychiatric disorders; (b) surgical operation on digestive tract, except appendectomy; (c) regular drug treatment within 15 days of the study; (d) inability to participate in the entire study; (e) inability to read and write; and (f) vigorous physical activity more than three times a week. It is important to mention that none of the participants engaged in any sports practice over the course of this investigation. The purpose of this investigation was to evaluate the UC hydration scale within children under their usual hydration process (i.e., not dehydrated due to water loss from exercise). Subjects were stratified by age and gender, and 15 boys and 15 girls were recruited for each age integer from 8 to 14 years old.

### Study procedure

During the first meeting, the selected subjects and their legal guardians were provided with detailed verbal and written instructions of the procedures of the study. Afterwards, children’s body weight and height without shoes and minimal clothing were recorded to the closet 0.1 kg and 0.01 m, respectively. Body composition and total body water (TBW) were estimated via hand-to-foot bioelectrical impedance analysis (Quantum, RJL, MI, USA). All subjects received financial compensation in the form of a gift certificate upon completion of their participation.

Subjects were instructed to collect all urine produced over 24 h, in provided opaque containers. Since the first-morning urine sample represents the fluid intake and activity of the day before, the subjects were instructed not to collect that sample, but instead, to collect the next day’s first sample. Three urine sample containers were provided: one individual container for the sample immediately before lunch (Noon), one individual container for the first-morning (AM) urine and a third container for all other voids. The first-morning and noon samples were analyzed individually, and then all samples were combined for the 24-h sample analysis. Subjects were also provided with a urine color scale [10] in order to match the color that best describes their urine color during the first-morning and noon urine samples. Subjects were briefed on the use of the urine color scale and instructed to urinate whenever their natural urge to use the bathroom dictated and to judge their urine color from the urine stream, not by looking at the color inside the container. Subjects were asked to provide only one color choice as a whole number integer. Numbers were assigned to the colors from 1, representing the lightest, to 8, representing the darkest. Urine containers were kept in an air-conditioned room and were picked up by the researchers within 24 h.

### Urine analysis

Urine samples were analyzed upon delivery to the laboratory and no later than 24 h after the collection. Osmolality was measured in duplicate, by freezing point depression (3D3 Advanced Osmometer, Advanced Instruments, Inc., MA, USA). Specific gravity was measured in duplicate using a handheld clinical refractometer (ATAGO SUR-NE, Tokyo, Japan). UC was determined from an experienced researcher by comparing the color of the urine sample placed in a clear, glass 15-mL tube against white background, under fluorescent lighting, next to an original urine color scale [31].

### Urinary definition of hypohydration

Hypohydration was defined as a UOsmo measurement ≥800 mmol kg^−1^ based on expert opinion and scientific research in a number of investigations [6, 11, 12]. Specifically, one highly relevant research study of German children, the analysis of 24-h urine samples, identified 830 mmol kg^−1^ as the threshold to identify hypohydration [13]. The second definition is not appropriate to this investigation because neither measurement of total body water nor intervention designed to manipulate total body water was used.

### Statistical analysis

The association of the urine color scale in young and healthy boys and girls as a marker of urine concentration (aim a) was tested by regression analysis, performed between the urine colors measured in the laboratory (Lab-UC-AM, Lab-UC-Noon, Lab-UC-24h) versus the corresponding urine osmolality (UOsmo-AM, UOsmo-Noon, UOsmo-24h). The correct classification ability of UC (aim b) was evaluated through the receiver operating characteristic (ROC) curve, performed after adjusting for several confounding variables (age, gender, and BMI). Threshold analysis was based on the ROC curve; the optimal value of UC to predict hypohydration (i.e., UOsmo ≥800 mmol kg^−1^) was revealed using the max–max approach of the sensitivity and specificity. Investigator- versus participant-evaluated UC values (aim c) were evaluated with the Bland–Altman comparison method [32]. Lab-UC-AM and Lab-UC-Noon ratings from trained investigators were compared with the children’s self-assessed UC rating at both the first-morning (Self-UC-AM) and mid-day (Self-UC-Noon) time points. For each of these variables, Spearman’s rho correlation coefficient was calculated between the difference in the two methods and their mean to assess whether the results were biased. Percentage of agreement was calculated as the ratio of participants who had mean differences between the two methods within the range [*M*_(difference)_ ± 1.96 SD_(difference)_]. A probability (*P*) level of 0.05 was defined for statistical significance. Statistical analyses were performed using SPSS (version 22, SPSS Inc., Chicago, IL, USA) and JMP Pro (version 11, SAS Inc., Gary, NC, USA).

## Results

Anthropometric characteristics of the 210 study participants are presented in Table 1, while mean values and ranges of measured hydration markers are presented in Table 2.

**Table 1:** Subject characteristics

	Mean ± SD	Range
Sample size (#)	210	210
Height (m)	1.49 ± 0.13	1.19–1.80
Weight (kg)	43.4 ± 12.6	21.4–82.0
BMI (kg m^−2^)	19.2 ± 3.2	13.2–32.8
Body fat (%)	25.2 ± 7.8	8.8–47.2
TBW (L)	25.9 ± 6.5	11.9–42.5

**Table 2:** Urinary hydration markers at different time points

	Mean ± SD	Range
Urine volume (mL)	1335 ± 620	545–4000
UOsmo-24h (mmol kg^−1^)	686 ± 223	261–1254
UOsmo-AM (mmol kg^−1^)	780 ± 235	263–1381
UOsmo-Noon (mmol kg^−1^)	747 ± 277	96–1302
USG-24h	1.018 ± 0.005	1.007–1.033
USG-AM	1.021 ± 0.006	1.008–1.038
USG-Noon	1.019 ± 0.007	1.002–1.035
Lab-UC-24h	2.9 ± 1.1	1–7
Lab-UC-AM	2.9 ± 0.9	1–6
Lab-UC-Noon	2.7 ± 1.0	1–5
Self-UC-AM	4.2 ± 1.3	1–7
Self-UC-Noon	3.6 ± 1.4	1–8

UOsmo, urine osmolality; USG, urine specific gravity; UC, urine color; AM, first morning; Noon, before lunch; Lab-UC, laboratory-assessed urine color; Self-UC, self-assessed urine color by the subjects

### Validity of the urine color scale to predict urine concentration

**Fig. 1:**
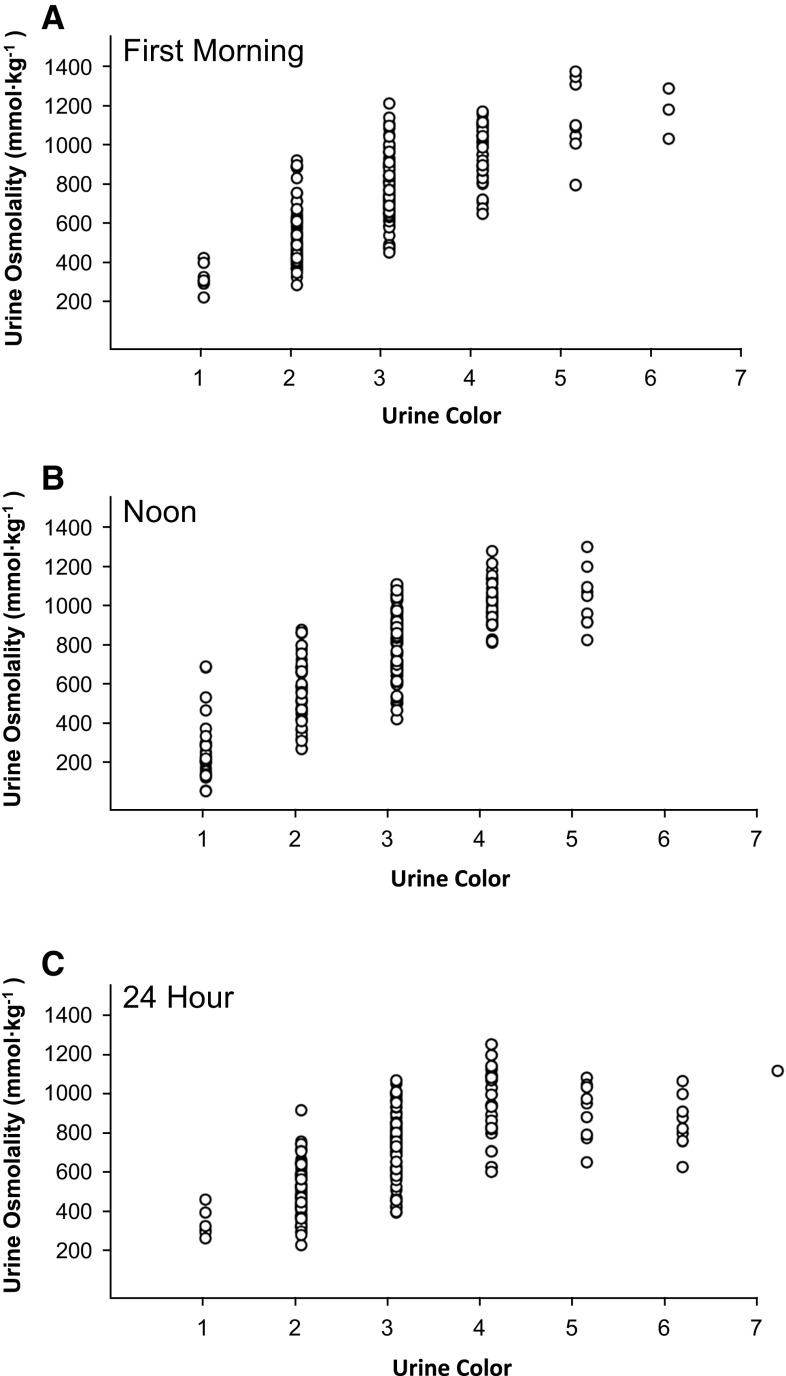
Interval regression analysis of urine color (UC) as a predictor of urine osmolality (UOsmo) from; **a** AM urine sample, **b** noon urine sample, **c** 24-h urine collection

Linear regression analysis revealed that UC was significantly associated with UOsmo for both of the single samples (Fig. 1a, b), as well as for the 24-h collection (Fig. 1c). UC ratings from first-morning samples (Lab-UC-AM) explained 56 % of the variance in UOsmo-AM (*β* = 187.9[11.5], *F*_[1,207]_ = 268.5, *P* < 0.001). Visually, the data do not seem to follow a linear trend, but trend analysis showed no significant differences between linear and nonlinear curves (quadratic, cubic, logarithmic, logistic and exponential). UC ratings from samples collected just before lunch (Lab-UC-Noon) explained 66 % of the variance in UOsmo-Noon (*β* = 217.0[11.3], *F*_[1,192]_ = 371.3, *P* < 0.001). Lastly, UC ratings from the complete 24-h collection (Lab-UC-24h) explained 45 % of the variance in Uosmo-24h (*β* = 136.4[10.6], *F*_[1,207]_ = 166.3, *P* < 0.001). Overall, UC displayed a strong positive relationship as a predictor of UOsmo in young and healthy children.

### Diagnostic value of urine color scale

ROC analyses defined the diagnostic ability of UC for hypohydration in the three separate laboratory-rated samples, after adjusting for age, gender, and BMI. UC maintained good diagnostic capability for identifying hypohydration indicative of urine osmolality ≥800 mmol kg^−1^ for both time points and the 24-h sample (Table 3; all *P* < 0.001). The term “good” is a rating used to describe the ability of the test (i.e., urine color) to detect the condition (i.e., hypohydration) when the area under the curve is between 0.80 and 0.89 [33]. The optimal threshold point for hypohydration in Lab-UC ratings was ≥3 (i.e., a sample rated as UC 3 or higher indicated hypohydration).

**Table 3:** Receiver operating characteristic evaluation of UC measured in the laboratory and self-assessment as a diagnostic tool for identifying hypohydration standard

Predictive variable	Diagnostic standard	Threshold	AUC	Sensitivity %	Specificity %
Lab-UC-24h	UOsmo-24h	3	0.90	98.4	59.6
Lab-UC-AM	UOsmo-AM	3	0.85	91.7	54.9
Lab-UC-Noon	UOsmo-Noon	3	0.92	94.6	68.3
Self-UC-AM	UOsmo-AM	4	0.67	88.0	34.6
Self-UC-Noon	UOsmo-Noon	4	0.74	70.4	71.1
Self-UC-AM	UOsmo-24h	4	0.68	87.5	29.7
Self-UC-Noon	UOsmo-24h	4	0.78	72.2	62.0

Predictive variable was tested against the corresponding hypohydration diagnostic standard UOsmo ≥800 mmol kg^−1^ from the listed sample 24-h, AM or Noon

### Self-assessment of urine color

The overall accuracy of the self-assessment of UC based on the morning or the noon sample ranged from 67 to 78 %. Self-assessment of the morning urine sample illustrated good ability to identify hypohydrated samples, but low ability to identify euhydration (Table 3). The diagnostic ability of the self-assessed UC of the morning and noon sample was tested in predicting overall 24-h hydration level. The area under the curve data from the Self-UC-AM and Self-UC-Noon samples indicated good diagnostic capability of identifying hypohydration indicative of urine osmolality >800 mmol kg^−1^ (Table 3; all *P* < 0.001). Further analysis indicated that the optimal self-assessed urine color threshold value for hypohydration was ≥4 (i.e., a self-assessed rating as UC 4 or higher indicated hypohydration).

**Fig. 2:**
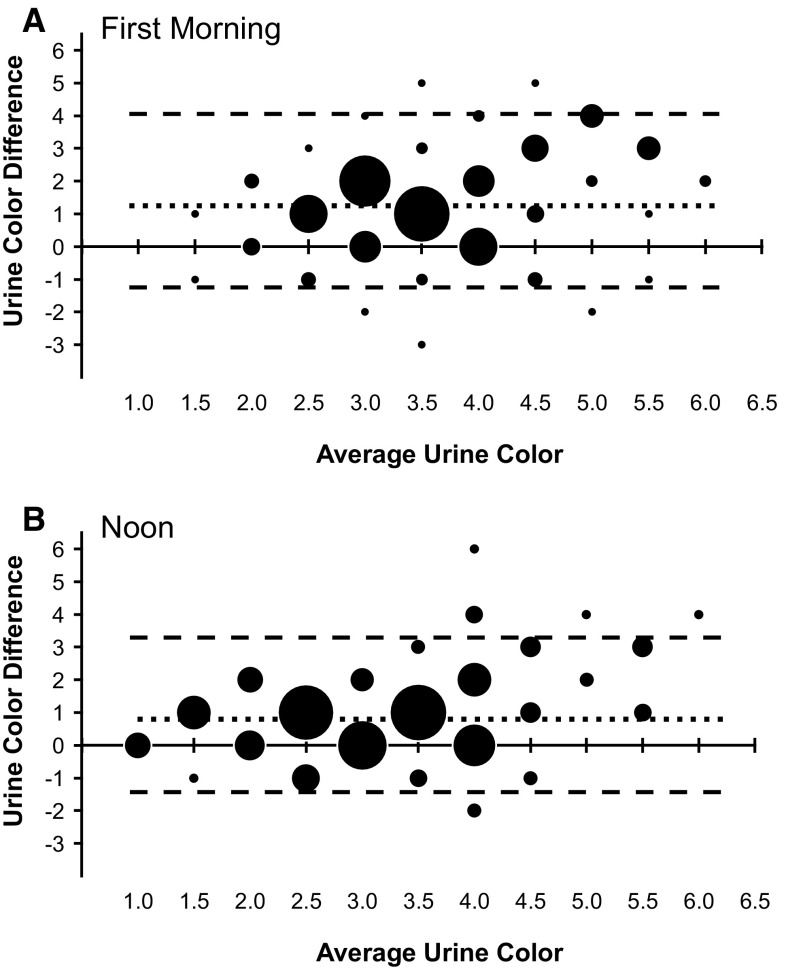
Bland–Altman analysis of self-assessed urine color versus laboratory-assessed urine color for individual urine samples collected at; **a** First-morning, AM time-point and, **b** noon time-point. The *x*-axis, average urine color, is the mean of each self-assessed and laboratory-assessed sample. The *y*-axis, urine color difference, represents the difference between laboratory-assessed and self-assessed urine color for each sample. The area of markers indicates the relative frequency of corresponding data point (i.e., *larger circles* designate more occurrences). *Upper and lower dashed lines* represent 95 % limits of agreement. *Middle dotted line* represents mean difference between respective Self-UC and Lab-UC

Bland–Altman analyses were employed to evaluate the agreement between self- and laboratory-assessed UC ratings for the first-morning (Fig. 2a) and noon (Fig. 2b) individual urine samples. There were two differences between self-assessed and laboratory-assessed samples. Understandably, the person evaluating the sample differed, and also self-assessment occurred with children being instructed to look at the urine stream, while laboratory-assessed was viewed with the sample in a 15-mL test tube. In this case, the Bland–Altman analysis is used to evaluate the agreement between two different methods of evaluating the same parameter (i.e., urine color). Because UC is an interval scale, many of the coordinates comparing mean UC rating versus UC rating difference occurred more than once (i.e., many data points for each dot). For example, only 30 markers represent data collected from all paired UC ratings in the first-morning figure. The frequency of each coordinate’s repetition is presented with the relative size (i.e., diameter) of each marker in Fig. 2. Additionally, because the *x*-axis on both figures represents the average of both ratings, values are displayed in 0.5 unit integers. This practice is avoided when comparing the index UC measurement against other dependent variables (i.e., Fig. 1). In this instance, the 0.5 integers allow for comparison of the measurement techniques and should not be interpreted as a UC that was evaluated to fall between two other integers. Self-UC ratings at both time points were similar compared to Lab-UC, as confirmed by levels of agreement of 93 and 96 % for UC-AM, and UC-Noon, respectively. However, the mean difference between self- and laboratory-assessed UC was 1.3 and 0.9 UC units for the morning and before-lunch sample, respectively. Additionally, Spearman’s Rho revealed that the results are slightly biased 0.267 and 0.267 (*P* ≤ 0.001) for each of the comparisons. These relationships showed that (1) individuals tended to rate UC darker than trained investigators, and (2) as UC increased, so did the discrepancy between the participant and investigator’s UC ratings.

## Discussion

The main findings of the current investigation were threefold. First, Lab-UC ratings from both individual urine samples and the 24-h collection were strongly positively associated with UOsmo in young and healthy children. Second, a laboratory-measured UC has a strong diagnostic capacity to identify hypohydration, and a UC of 3 or greater is indicative of hypohydration in individual and 24-h urine samples. Finally, self-assessed UC has reasonable ability to diagnose hypohydration with a UC of 4 or greater. Thus, the data indicate that urine color is a valid method for hydration self-assessment in children. To our knowledge, this is the first study showing that the original eight-point urine color scale [10] is a valid assessment technique in children.

These findings expand the applicability of the urine color scale to include application within free-living children. The initial urine color investigations approximately 20 years ago, introduced the color scale through a series of experiments performed in adult athletes (mostly males) during vigorous physical activity in the heat, which induced body water deficits [10]. In this report, the validity of UC was established by observed strong positive relationships and similar frequency distributions of UC ratings in comparison with previously established hydration markers (i.e., UOsmo and USG). A follow-up study validated the urine color scale against changes in body water during dehydration, exercise and rehydration [9]. This investigation showed that UC is valid through visual inspection of changes in UC, which paralleled the changes of body water change across different phases of de- and rehydration. Outside of exercise, UC has shown to be different between adults habitually consuming low or high fluid volumes in normal daily living conditions [16], and to respond to changes in daily fluid intake [30], both in 24-h and shorter urine collections. Our results support these previous findings by replicating the strong positive relationship between UC and UOsmo, and by indicating that UC can robustly identify hypohydration in children. Additionally, this is the first investigation to validate the urine color scale separate from acute body water loss in children.

The diagnostic ability of urine color for hypohydration was examined, and a threshold value of UC ≥3 was determined for laboratory-assessed urine color. In the original study, the authors suggested that UCs of 1, 2 and 3 indicated that an athlete is well hydrated, and within 1 % of their baseline body mass. However, this differs from the intricate work of Cheuvront and colleagues [34] who calculated a UC threshold value of 5.5 to diagnose a body mass loss of 2 % or greater following 3–5 h of exercise-induced dehydration. The exercise employed to induce dehydration is sure to have had an impact on the hydration process and the response of urinary hydration markers, as has previously been observed [35]. Thus, we are not suggesting that an observed UC of ≥5.5 is not indicative of dehydration after beginning exercise in a euhydrated state. Instead, the present investigation suggests that a UC ≥3 is indicative of body water conservation and hypohydration in non-athlete children during everyday living. These two diagnoses are both related to body water content but must not be confused to be equivalent.

A lower observed UC threshold value could also be attributed to the method of assessment. In the original studies, UC was assessed, “by holding each specimen next to the color scale” [10]. In the present study, investigators transferred the sample to a 15-mL clear glass tube prior to holding it adjacent to the scale. The Beer–Lambert law states that light absorbance is equal to the product of, the concentration of the solution the light is passing through, the length of the solution the light passes through, and the absorption coefficient. Thus, two physical factors of the sample container, the diameter of the urine cup (smaller vs. larger) and the material of the container (glass vs. plastic), can affect the amount of light absorbed by the sample/container combination, potentially influencing the color rating as the light reaches the investigators eye. Due to the smaller diameter of the test tube compared with a standard urine specimen cup and the clarity of glass versus a plastic cup, it is possible that the UC values presented above could have been shifted toward lower ratings (i.e., lighter color). However, a clear glass test tube avoids potential artifact as it allows the least obstructed view of the sample and it is standard piece of laboratory supply that can be found in everywhere in the world. As one of the above-stated aims was to identify hypohydration, this manner of sample preparation was employed to ensure other laboratories would be able to easily replicate the listed methodology.

The simplicity of the UC scale lends itself impeccably to self-assessment. However, previous studies of self-assessment of hydration status have only been related to exercise and knowledge of body water loss versus fluid consumption [36]. Our data indicate that in a free-living population of children, self-assessment of UC has a good ability to identify hypohydration. The ROC analysis indicated a self-assessed UC threshold value of 4 or greater to be consistent with hypohydration. The 1 integer elevation from the laboratory UC threshold is confirmed through the agreement plots that verify self-assessments tended to be ~1 integer higher than laboratory-judged UC ratings (Fig. 2). The fact that the children had never used the urine color scale before could be a potential reason for the overestimation. The investigators gave appropriate instruction, familiarized each participant with the urine color scale and stated that they should judge their color rating while evaluating the urine stream. Based on the above discussion of light absorbance through liquids, this overestimation by the children is counter-intuitive. The narrower stream of urine absorbs less light compared to the investigator-viewed test tube, which could have explained if lower UC ratings were reported through self-assessment. However, we believe the discrepancy most likely occurred because self-assessment took place with the children looking down, minimizing potential light sources behind the sample. Therefore, less light was available to pass through the sample, shifting the self-assessments toward the darker range of the UC scale.

One further explanation of the discrepancy between laboratory- and self-assessed UC ratings is related to overall perception of color. A benefit of the UC scale is that readings should not be impacted by typical color blindness which limits sensitivity to red, green or blue/violet light [37]. However, age has been shown to play a role in color perception, with individuals between the age of 20–50 years best able to properly identify colors and discriminate between hues [38]. Additionally, children can find discrimination of hues with low saturation difficult [39]. These differences in color perception between age groups could explain why children tended to rate urine color higher and why the agreement between self- and laboratory-assessed UC was biased, (i.e., the difference between the UC ratings increased as mean UC increased). Thus, we recommend that self-assessed UC only be used to differentiate between euhydrated and hypohydrated. When the full integral scale is needed (i.e., when it is important to differentiate between UC 4 and 5), laboratory-assessed UC is a superior technique.

Lastly, to further examine the ability of young boys and girls to self-diagnose hypohydration based on the individual time-point urine color, we performed ROC analysis of self-assessed single UC ratings from morning and before-lunch samples against 24-h UOsmo. The results from the present study demonstrated that both Self-UC-AM and Self-UC-Noon demonstrated high overall sensitivity with respect to UOsmo-24h, and therefore, either can be recommended as a diagnostic tool for whole-day hydration assessment. However, from a practical point of view, this finding provides substantial advantages as the 24-h urine collection procedure and the first-morning sample present several obstacles as far as relation to free-living data collection. First, the collection of a 24-h urine sample is an impractical method, especially for children who are engaged in a variety of activities throughout the day. Additionally, the use of the first-morning urine sample for the assessment of hydration status has been documented as susceptible to errors, leading to a slightly higher estimation of hypohydration [40, 41]. Hence, we suggest that UC self-assessment of a mid-day sample is most preferable as a valid and simple estimate of whole-day hydration status.

Overall, the UC scale has produced more than 40 peer-reviewed and PubMed-indexed articles relating its use to distinguishing hydration status. However, in comparison with more established hydration markers such as body mass, UOsmo or plasma osmolality, the depths of the findings are only beginning to scratch the surface. In order to progress with any measurement, it is integral to first establish its validity in the most controlled environments. We feel that the above data have achieved this task by testing the urine color scale free from the influence of exercise or acute body water loss. In young and healthy Greek boys and girls, UC is a valid method for assessing urine concentration and is capable of discriminating between eu- and hypohydration, and self-assessment of urine color could become a favorable practical hydration status marker.

## References

[1] Thornton SN (2010). Thirst and hydration: physiology and consequences of dysfunction. Physiol Behav.

[2] Bardis CN, Kavouras SA, Kosti L, Markousi M, Sidossis LS (2013). Mild hypohydration decreases cycling performance in the heat. Med Sci Sports Exerc.

[3] Sontrop JM, Dixon SN, Garg AX, Buendia-Jimenez I, Dohein O, Huang SH, Clark WF (2013). Association between water intake, chronic kidney disease, and cardiovascular disease: a cross-sectional analysis of NHANES data. Am J Nephrol.

[4] Michaud DS, Spiegelman D, Clinton SK, Rimm EB, Curhan GC, Willett WC, Giovannucci EL (1999). Fluid intake and the risk of bladder cancer in men. N Engl J Med.

[5] Kavouras SA, Arnaoutis G, Makrillos M, Garagouni C, Nikolaou E, Chira O, Ellinikaki E, Sidossis LS (2012). Educational intervention on water intake improves hydration status and enhances exercise performance in athletic youth. Scand J Med Sci Sports.

[6] Bar-David Y, Urkin J, Kozminsky E (2005). The effect of voluntary dehydration on cognitive functions of elementary school children. Acta Paediatr.

[7] Landau D, Tovbin D, Shalev H (2000). Pediatric urolithiasis in southern Israel: the role of uricosuria. Pediatr Nephrol.

[8] Baron S, Courbebaisse M, Lepicard EM, Friedlander G (2014) Assessment of hydration status in a large population. Br J Nutr 1–12 10.1017/S0007114514003213 25418739

[9] Armstrong LE, Soto JA, Hacker FT, Casa DJ, Kavouras SA, Maresh CM (1998). Urinary indices during dehydration, exercise, and rehydration. Int J Sport Nutr.

[10] Armstrong LE, Maresh CM, Castellani JW, Bergeron MF, Kenefick RW, LaGasse KE, Riebe D (1994). Urinary indices of hydration status. Int J Sport Nutr.

[11] Bar-David Y, Urkin J, Landau D, Bar-David Z, Pilpel D (2009). Voluntary dehydration among elementary school children residing in a hot arid environment. J Hum Nutr Diet.

[12] Fadda R, Rapinett G, Grathwohl D, Parisi M, Fanari R, Calo CM, Schmitt J (2012). Effects of drinking supplementary water at school on cognitive performance in children. Appetite.

[13] Manz F, Wentz A, Sichert-Hellert W (2002). The most essential nutrient: defining the adequate intake of water. J Pediatr.

[14] Manz F, Wentz A (2003). 24-H hydration status: parameters, epidemiology and recommendations. Eur J Clin Nutr.

[15] Stookey JD, Brass B, Holliday A, Arieff A (2012). What is the cell hydration status of healthy children in the USA? Preliminary data on urine osmolality and water intake. Public Health Nutr.

[16] Perrier E, Vergne S, Klein A, Poupin M, Rondeau P, Le Bellego L, Armstrong LE, Lang F, Stookey J, Tack I (2013). Hydration biomarkers in free-living adults with different levels of habitual fluid consumption. Br J Nutr.

[17] Costill DL, Cote R, Fink W (1976). Muscle water and electrolytes following varied levels of dehydration in man. J Appl Physiol.

[18] Nose H, Mack GW, Shi XR, Nadel ER (1988). Shift in body fluid compartments after dehydration in humans. J Appl Physiol (1985).

[19] Ehrig F, Waller S, Misra M, Twardowski ZJ (1999). A case of ‘green urine’. Nephrol Dial Transplant.

[20] Sands JM, Layton HE (2009). The physiology of urinary concentration: an update. Semin Nephrol.

[21] Linderkamp O, Versmold HT, Riegel KP, Betke K (1977). Estimation and prediction of blood volume in infants and children. Eur J Pediatr.

[22] Weil JV, Jamieson G, Brown DW, Grover RF (1968). The red cell mass–arterial oxygen relationship in normal man. Application to patients with chronic obstructive airway disease. J Clin Invest.

[23] EFSA Panel on Dietetic Products, Nutrition, and Allergies (2010). Scientific opinion on dietary reference values for water. EFSA J.

[24] Food and Nutrition Board, Institute of Medicine (2004). Dietary reference intakes for water, potassium, sodium, chloride, and sulfate.

[25] Drewnowski A, Rehm CD, Constant F (2013) Water and beverage consumption among children age 4–13y in the United States: analyses of 2005–2010 NHANES data. Nutr J 12:85-2891-12-85 10.1186/1475-2891-12-85 PMC3698018 23782914

[26] Park S, Blanck HM, Sherry B, Brener N, O’Toole T (2012). Factors associated with low water intake among US high school students—National Youth Physical Activity and Nutrition Study, 2010. J Acad Nutr Diet.

[27] Bonnet F, Lepicard EM, Cathrin L, Letellier C, Constant F, Hawili N, Friedlander G (2012). French children start their school day with a hydration deficit. Ann Nutr Metab.

[28] Shephard MD, Penberthy LA, Fraser CG (1981). Short- and long-term biological variation in analytes in urine of apparently healthy individuals. Clin Chem.

[29] Armstrong LE, Pumerantz AC, Fiala KA, Roti MW, Kavouras SA, Casa DJ, Maresh CM (2010). Human hydration indices: acute and longitudinal reference values. Int J Sport Nutr Exerc Metab.

[30] Perrier E, Demazieres A, Girard N, Pross N, Osbild D, Metzger D, Guelinckx I, Klein A (2013). Circadian variation and responsiveness of hydration biomarkers to changes in daily water intake. Eur J Appl Physiol.

[31] Armstrong LE HydrationCheck. In: Human hydration, LLC. http://www.hydrationcheck.com/index.php. Accessed 10/2/2014

[32] Bland JM, Altman DG (1986). Statistical methods for assessing agreement between two methods of clinical measurement. Lancet.

[33] Tape T The area under an ROC curve. In: interpreting diagnostic tests. University of Nebraska Medical Center. http://gim.unmc.edu/dxtests/ROC3.htm. Accessed 03/04/2015

[34] Cheuvront SN, Ely BR, Kenefick RW, Sawka MN (2010). Biological variation and diagnostic accuracy of dehydration assessment markers. Am J Clin Nutr.

[35] Munoz CX, Johnson EC, Demartini JK, Huggins RA, McKenzie AL, Casa DJ, Maresh CM, Armstrong LE (2013) Assessment of hydration biomarkers including salivary osmolality during passive and active dehydration. Eur J Clin Nutr 10.1038/ejcn.2013.195 24129362

[36] Decher NR, Casa DJ, Yeargin SW, Ganio MS, Levreault ML, Dann CL, James CT, McCaffrey MA, Oconnor CB, Brown SW (2008). Hydration status, knowledge, and behavior in youths at summer sports camps. Int J Sports Physiol Perform.

[37] Jeffries BJ (1880). On color blindness. Science.

[38] Roy MS, Podgor MJ, Collier B, Gunkel RD (1991). Color vision and age in a normal North American population. Graefes Arch Clin Exp Ophthalmol.

[39] Gaines R (1972). Variables in color perception of young children. J Exp Child Psychol.

[40] Hamouti N, Del Coso J, Avila A, Mora-Rodriguez R (2010). Effects of athletes’ muscle mass on urinary markers of hydration status. Eur J Appl Physiol.

[41] Oppliger RA, Magnes SA, Popowski LA, Gisolfi CV (2005). Accuracy of urine specific gravity and osmolality as indicators of hydration status. Int J Sport Nutr Exerc Metab.

